# Control Systems with Tomographic Sensors—A Review

**DOI:** 10.3390/s22082847

**Published:** 2022-04-07

**Authors:** Jaroslav Hlava, Shereen Abouelazayem

**Affiliations:** Institute of Mechatronics and Computer Engineering, Faculty of Mechatronics, Informatics and Interdisciplinary Studies, Technical University of Liberec, 461 17 Liberec, Czech Republic; shereen.abouelazayem@tul.cz

**Keywords:** industrial process tomography, process control, control systems in process engineering, distributed parameters systems

## Abstract

Industrial process tomography offers two key advantages over conventional sensing systems. Firstly, process tomography systems provide information about 2D or 3D distributions of the variables of interest. Secondly, tomography looks inside the processes without penetrating them physically, i.e., sensing is possible despite harsh process conditions, and the operation of the process is not disturbed by intrusive sensors. These advantages open new perspectives for the field of process control, and the potential of closed-loop control applications is one of the main driving forces behind the development of industrial tomography. Despite these advantages and decades of development, closed-loop control applications of tomography are still not really common. This article provides an overview of the current state-of-the-art in the field of control systems with tomographic sensors. An attempt is made to classify the different control approaches, critically assess their strengths and weak points, and outline which directions may lead to increased future utilization of industrial tomography in the closed-loop feedback control.

## 1. Introduction

Industrial process tomography is a relatively new development in the field of sensor systems. Industrial applications of tomography were reported in the 1970s; however, they mostly used ionizing radiation, and hence they were not suitable for normal industrial use [1]. Not only was there an obvious safety issue, but most of these tomography sensors required very long times to collect the data and reconstruct the tomograms. These times were in the range of several seconds to several hours or even more according to a review paper published in 1984 [2]. For this reason, they were suitable for purposes such as quality control, and detection of cracks and other defects. However, they could not be used for real-time closed-loop control. The actual origins of industrial tomography can be dated back to the mid-1980s when several laboratories started the development of tomography systems that were not based on electromagnetic radiation but used measurements of various electrical properties (e.g., electrical capacitance tomography electrical, impedance tomography, see [1] for these early developments).

These electric-based tomography systems reached a certain degree of maturity in the 1990s. Compared to tomography using ionizing radiation, they were more suitable for industrial applications. This was due to their safety and their faster response times. Towards the end of the 1990s, these sensors achieved scanning speeds in the range of several hundreds of frames per second [3]. Modern electric-based tomography sensors achieve even higher speeds in the range of thousands of frames per second up to about 10,000 frames per second in the case of wire mesh sensors [4,5].

It is generally known that industrial process tomography can be used as a super-sensing technology that allows us to see inside industrial processes. This ability to look inside the process has an obvious advantage: it creates a cross-sectional or volumetric image of the internal physical properties of an object, i.e., the measurement data are richer in information than single-point measurements [6]. Moreover, most (though not all) tomography modalities look inside the processes without penetrating them physically [7]. This ability is advantageous if the conditions inside the process are harsh (e.g., high temperatures, high pressures, corrosive environments). Nonintrusive sensors are also necessary for processes that might be disturbed by intrusive sensors (e.g., the flow in a fluid separator would be disturbed and separation efficiency decreased by an intrusive sensor in the region where fluids are separated) [8].

Considering these advantages of industrial tomography systems, it is not surprising that the applications of process tomography for closed-loop real-time control have been one of the main stated purposes of the development of process tomography since its very beginning. The focus of the present paper is on closed-loop control using the measurements from tomographic sensors. Hence, it is definitely not its intention to give a detailed treatment of the development of industrial tomography. However, it will be helpful to introduce the major monographs on industrial tomography published at intervals of one decade and see how they view the relationship between tomography and closed-loop control.

In 1995 an extensive monograph, *Process Tomography—Principles, techniques and applications*, edited by R. A. Williams and M. S. Beck was published [9]. Its focus on automatic control is more than evident. Descriptions of tomography modalities and tomography applications are mostly concluded with explicit references to automatic control as one of the primary purposes for process tomography. “Process monitoring and control in an industrial environment” is one of the five main categories of applications of process tomography in this monograph. The next major monograph on process tomography was published ten years later in 2005, titled: *Process Imaging for Automatic Control* [10]. In this monograph, tomography is categorized under the more general term of imaging sensors, and the main focus on automatic control is explicit even in the monograph title.

On the contrary, if we make another decade jump in time to 2015 we have a different picture. An even more extensive monograph called, *Industrial Tomography Systems and Applications* [11] was published in this year. It is interesting to note that, although the application part of this monograph is quite long (about 40% of its total length), there are minimal references to control. Closed-loop control applications of tomography are missing almost completely. A similar impression appears when looking at the last published proceedings of the World Congresses on Industrial Process Tomography [12]. Each of the proceedings of the last three congresses (2016, 2018, and 2021) includes only one paper describing a closed-loop control application of tomography [13,14,15]. Moreover, papers [13,14] use hard-field tomography, which is less standard in industrial applications, and Ref. [15] describes a closed-loop control application that is rather biomedical than industrial (control of mechanical ventilation based on lung electrical tomography). This contrasts with the first congresses (1999, 2001, 2003) where tomography-based control played a much more significant role.

Major monographs and proceedings of the World Congresses on Industrial Process Tomography represent just a fraction of works related to process tomography. Section 3 of the present paper offers a more exhaustive review of the published papers on closed-loop control based on tomographic sensors. Nevertheless, as we will see, the overall trend is the same. It is important to note that this relative decline in the number of published papers concerns only papers describing complete closed-loop control applications of tomography. Industrial tomography itself is a subject of keen research interest. The number of papers focused on advances in the design of process tomography sensors and tomography data processing is constantly growing. Moreover, these advances are often connected with more or less explicit statements that closed-loop control is their ultimate purpose [4,16,17,18,19,20].

In the end, this leaves an ambivalent impression. All building blocks for creating a tomography-based closed loop are available. They enjoy unceasing and growing research attention, and they are much more advanced than two decades ago. The only missing thing is a corresponding development of closed-loop control applications.

Hence the present paper will have a twofold purpose. Firstly, and most importantly, there is no up-to-date, clear description of the current state-of-the-art in the field of tomography-based control. The last publication that reviews control systems with tomography sensors is the monograph, *Process Imaging for Automatic Control* [10] published in 2005, i.e., 17 years ago. The more recent results are scattered in various conferences and journal papers, and no review papers exist. The purpose of the present review paper is to fill this gap and survey the current state-of-the-art in the field of control systems based on tomographic sensors. The focus will be on papers that describe complete closed-loop applications, while we will mostly put aside those numerous papers that deal only with tomography data processing or tomography hardware. We will concentrate on the developments after 2005 with occasional excursions more to the past because they are often necessary to understand the development of the main concepts. Secondly, we would like to analyze and critically assess the strengths and weak points of the various automatic control approaches used in connection with tomographic sensors and outline which directions may lead to increased future utilization of industrial tomography in closed-loop control.

The paper is organized as follows. Section 2 briefly discusses control-related properties of tomographic sensors, mainly their distributed sensing property and processing of sensor data. Processing of the measurements from the tomographic sensors is a vast subject by itself. It is definitely not the purpose of the present paper to give a detailed treatment. The focus is only on the control implication of various approaches to working with data from tomographic sensors. Section 3 is the main section of this paper. It focuses on the various control techniques used with tomographic sensors and their application areas. The emphasis is on automatic control methods. Section 3 is therefore divided into subsections according to this principle. For this reason, some application areas are mentioned several times because similar processes were controlled using different methods by various research groups. Section 4 summarizes and discusses the state-of-the-art described in Section 3 and outlines future research directions.

## 2. General Characteristics of Tomography-Based Control

### 2.1. Implications for Selection of Control Method

Automatic control theory includes no methods and techniques developed specifically with the objective to be used in connection with tomographic sensors. Nevertheless, tomographic sensors have specific features that make some methods preferable to others. Additionally, standard control methods must often be modified or extended to be used in connection with tomography. For this reason, we will first give a general overview of control relevant characteristics of tomographic sensors before going to more specific descriptions of their control applications.

Tomographic sensors can look inside the industrial processes. This ability to look inside means in the first place that the data provided by tomographic sensors are multidimensional. The controller can perceive the variable(s) of interest within the whole plane or volume inside the process [4]. This distributed sensing fits well with the fact that there are many industrial processes whose state cannot be adequately characterized by a limited set of single-point measurements. Many such processes will later be described in greater detail. However, even without a detailed description, it is clear that, e.g., single-phase/multi-phase flow processes are characterized by velocity fields, concentrations, particle moistures, flow patterns, and other similar characteristics which are always defined in the whole volumes or cross-sections of the process not just at single points.

Single-point measurements correspond to ordinary differential equation (ODE) process models. A first-order differential equation can describe the dynamics of each single-point variable, and the complete model is then in the form of a set of a finite number of first-order differential equations. This form of the model is called a state-space model [21]. In its simplest linear timer invariant form, such a model can be written as

(1) $$\begin{array}{c} \frac{dx\left(t\right)}{dt}=Ax(t)+Bu(t)\\ y(t)=Cx(t)+Du(t) \end{array}$$

where ***x***(t) is an *n*-dimensional state vector

(2) $$x\left(t\right)=\left[\begin{array}{c} x_{1}\left(t\right)\\ x_{2}\left(t\right)\\ \vdots \\ x_{n}\left(t\right) \end{array}\right]$$

Its *n* scalar components (single-point variables) are called state variables. Vector ***u***(*t*) is a vector of input variables, ***y***(*t*) is a vector of output variables, ***A***, ***B***, ***C*** and ***D*** are matrices of appropriate dimensions. The number of first-order equations *n* is finite, and it is called the order of this model.

On the contrary, if the variables of interest are not located in a finite number of points but are distributed within a whole plane or volume, model (1) is no longer adequate. Its order would have to be infinity. Although it would be possible to construct a model with a structure similar to (1), ***A***, ***B***, ***C***, ***D*** would be infinite-dimensional operators on Hilbert spaces and not matrices [22]. Such models are popular in textbooks on the mathematical theory of infinite-dimensional systems. However, their relationship to physics is indirect and their practical applicability is more than limited. Nevertheless, they are closely related to partial differential equations (PDEs) and can be derived from them [22]. For this reason, it is better to work with models in the form of PDEs that can be derived from the underlying physics of the process.

Control of PDE systems is a specific branch of control theory with a relatively long history. Its origins date back to the 1960s or even earlier [23]. Now this theory has reached a considerable degree of maturity. Many textbooks are available, and a plethora of control methods has been developed. References [24,25,26] can serve as good introductory texts for PDE control and [27] for state estimation. However, they are just a small selection from a substantially larger body of the existing literature. Considering the distributed sensing property of tomographic sensors and advanced development of the PDE systems theory, tomography and control theory of PDE systems seem to be the most natural connection. Models and sensors are here consistent with each other. We will see in the next section to what extent this is really the case.

Nowadays, controllers are implemented mainly using digital computers. An implementation based on analog electronics is also possible but much less common [28]. Any nontrivial controller is also a dynamical system. If it is linear, it can be described by matrix Equation (1). In these equations, the derivative of each component of the state vector is expressed as a linear combination of state variables and input variables. Therefore, one integrator (analog or digital) is necessary to implement each row of these equations [21]. For this reason, it is clear that regardless of whether the controlled process itself is described by ODEs (i.e., it is finite-dimensional) or PDEs (i.e., it is infinite-dimensional), the controller must always be finite-dimensional. Otherwise, it would not be technically implementable because it would need infinitely many integrators. However, the transition to finite-dimensional description (if needed, i.e., with PDE process models) can be done at different design stages. Consequently, all design methods for control of PDE systems can be classified either as early lumping methods or late lumping methods, depending on when this transition is done.

Early lumping methods use approximation and model reduction techniques as a first step. PDE systems are reduced to finite-dimensional descriptions before controller design using the finite difference method, finite element method (FEM), or other techniques [29]. Regardless of which technique is used, the result is a state-space model, whose order may be relatively high, but still, it is finite.

This early lumping procedure may be illustrated using a simple example of a PDE model representing a forced-flow steam-jacketed tubular heat exchanger [30]. The space coordinate is one dimensional in the direction of the heat exchanger tube (*x*-axis). The dynamic model of this process can be expressed as:

(3) $$\frac{\partial T\left(x,t\right)}{\partial t}=-u\frac{\partial T\left(x,t\right)}{\partial x}+\frac{1}{\tau }\left(T_{j}\left(t\right)-T\left(x,t\right)\right)$$

where *T*(*x*,*t*) is the temperature of the heated liquid in the heat exchanger tube, *T_j_*(*t*) is the steam jacket temperature, *u* is the heated fluid velocity through the tube, and *τ* is a positive constant. The steam temperature is considered to be spatially uniform for simplicity.

If the heat exchanger is space discretized into *N* discrete elements of equal length Δ*x*, the space derivative can be approximated using the backward difference method

(4) $$\frac{\partial T_{L}}{\partial x}\approx \frac{T\left(i\right)-T\left(i-1\right)}{\Delta x}$$

Substituting the formula for backward difference into Equation (3), the following model is obtained

(5) $$\frac{dT\left(i,t\right)}{dt}=-\frac{u}{\Delta x}T\left(i-1,t\right)-\left(\frac{u}{\Delta x}+\frac{1}{\tau }\right)T\left(i,t\right)+\frac{1}{\tau }T_{j};\;i=1,\ldots ,N$$

where *T*(*i*,*t*) are temperatures of individual discrete elements, and *T*(0,*t*) is the input temperature. It is evident that if the state vector ***x*** is composed of temperatures *T*(1,*t*) to *T*(*N*,*t*), input vector ***u*** includes *T*(0,*t*) and *T_j_*(*t*), and output from the system is *T*(*N*,*t*), the set of Equation (5) can be written in matrix form (1).

If the model is in the form (1), i.e., in the standard linear state-space form, controller design can also proceed in a standard way and the number of available design methods and tools is very high. These methods include classical approaches based on state feedback such as the Linear Quadratic (LQ) control as well as modern advanced approaches based on numerical optimization such as Model Predictive Control (MPC) [31]. However, the reduction of infinite-dimensional to finite-dimensional dynamics implies that some part of the original dynamics is neglected. This may result in the so-called spillover effect [32]. This effect is marked by bad control performance or even closed-loop instability if the controller designed on the basis of the reduced lumped parameters model is connected to the real plant.

On the other hand, the late lumping approach exploits the full PDE model and uses the PDE systems theory for controller design. Lumping (in principle similar to the procedure outlined above in Equations (3) to (5)) is the last step to be done to implement the controller. Late lumping introduces no approximations during controller design, but the whole procedure requires a significantly deeper knowledge of PDE system theory [25].

The advantages of tomographic sensors are not limited to the fact that their sensing is distributed. Their ability to look inside the process also has the aspect that most of their modalities are nonintrusive [7]. They look inside the processes without penetrating them. Since most of the process tomography modalities are non-optical, they can be used even if the processes contain opaque fluids and the conditions inside them are very harsh. For these reasons, tomographic sensors can provide information that is not obtainable in other ways [4]. In the following pages of this review, we will see that incorporating tomographic sensors in feedback control makes sense even if their distributed sensing capability is not fully utilized, but their measurements are condensed into one or a few simple numerical values characterizing the behavior of the controlled process.

### 2.2. Tomography Data Processing

A very important general consideration related to the use of tomographic sensors for feedback control is the fact that they do not simply provide an image of what is inside the process. Their raw output is a set of measurements. The image must be reconstructed from this output. The reconstruction procedure depends on the specific tomography modality used. Medical tomography usually belongs to the so-called hard-field tomography category (e.g., X-ray, gamma-ray, ultrasound tomography). In this case, the path of the transmitted signal is a straight line, and signal strength is affected only by the material along that path. On the other hand, industrial process tomography usually belongs to the category of the so-called soft-field tomography (e.g., electrical impedance, electrical capacitance, magnetic induction tomography). In the soft-field-tomography, the path of the signal is not simply a straight line but there are other factors that can influence the transmitting signal (e.g., the distribution of conductivity or permeability inside and outside the measured region in the case of ERT or ECT) [6].

The nature of soft-field tomography is more complex. The reconstruction of the image from raw measurement data poses an increased difficulty. Most importantly, it is an ill-posed and often nonlinear problem in the case of most soft-field tomography modalities. It is not the purpose of this paper to give an overview of image reconstruction methods. An interested reader can find a good survey in [11] Part two, “Tomographic image reconstruction”, as well as in [33].

A more important point from the perspective of control applications is the fact that it is not always necessary to proceed in the seemingly most natural way: *raw measurement data* *→ image reconstruction resulting in a pixel-based image* *→ image analysis and interpretation where some control relevant features are extracted* *→ feedback control*. Following [34,35], we can say that there are at least two alternatives to full image reconstruction. Firstly, raw measurement data may often provide enough information for feedback control in many applications. Secondly, even if an image reconstruction step is performed, it need not be a blind reconstruction assuming that any image can arise from measurements. Rather it should incorporate prior information about the process into the reconstruction of the images.

Approaches relying on the analysis of raw data have been used since the beginning of process tomography. In [36], principal component analysis (PCA) was used on the raw data to relate the material concentration to the principal components of the raw data. Therefore, calculating the phase concentration of the two-phase flow was possible without reconstructing the image. Another early example is [37], where the time topology of flow was recreated by plotting signals corresponding to the peripheral impedance measurements, thus avoiding the reconstruction of the image. In [38] the flow regime identification was performed on the basis of the so-called fingerprint matching method. This method uses a 12-electrode electrical capacitance tomography sensor, i.e., with 66 capacitance measurements. For any specific flow regime, the data present a particular pattern. Such typical patterns for various flow regimes were stored in memory. The flow regime could then be identified by comparing the currently measured pattern with the set patterns stored in the memory.

The flow-pattern identification method described in [39] used two simple numerical identifiers obtained directly from electrical capacitance tomography measurements: (1) variance of on the electrode pairs of the same type (facing as well as first, second, and third adjacent electrodes) and (2) the ratio of the capacitance value for the top electrode pairs to the bottom electrode pairs. Based on these identifiers, it was possible to classify different variants and combinations of annular flow and stratified flow patterns. Although paper [39] is focused on flow pattern identification only, and its authors do not go on to consider control, it should be noted that the output from this classification is not a numerical continuous-valued variable but a set of discrete flow patterns with some intermediate combinations between them. Such a situation is not uncommon with tomography sensors. It is not well suited for using classical feedback controllers that need continuous-valued set-points and controlled variables. However, it might be well suited for using fuzzy valued variables and fuzzy control where a knowledge base can be utilized using prior knowledge of the flow patterns.

A similar but more recent example of the use of raw data is described in [40]. In this paper, the twin-plane ECT was used to sense two-phase flow involving water, air, and particulate flow in a fluidized bed. There was no image reconstruction. The raw capacitance data from each frame were processed using several alternative approaches, most importantly using neural networks with deep learning and cascades of Support Vector Machines (SVM). Similarly, as in [39], the result was a classification of the flow into several categories of flow patterns. Compared to [39], this classification was somewhat finer: plug, slug, annular, stratified, and wavy flow.

Regarding the second alternative to pixel-based image reconstruction, it can be said that there have been two common methodologies for the incorporation of prior information with the reconstruction of the images: Parametric modeling and state estimation. Parametric modeling makes use of the known geometry of the problem. Its principle can be explained using the hydrocyclone application described in [34]. Hydrocyclones form a central air core extending over the full length of the hydrocyclone. This core has a circular shape. Hence if the objective is to identify this core and find its diameter, we have a problem with circular geometry. For any given diameter of the core, we can calculate theoretically which tomographic measurements should be expected. The research described in [4,34] used ultrasound tomography. The air core diameter was found by searching for such diameter of the circle that produced calculated data that would be the best fit for the measured data.

**Figure 1:**
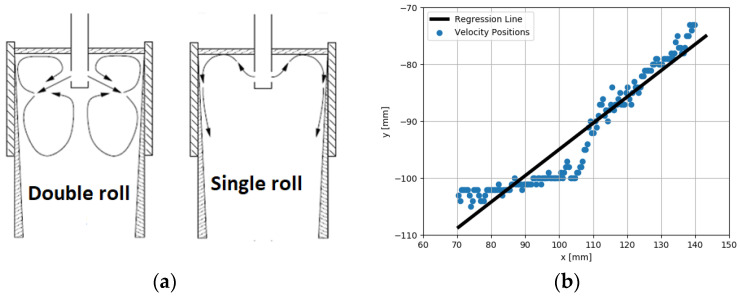
(**a**) Flow patterns in the mold; (**b**) parametrization of the jet flow using a line with a variable angle.

Parametric modeling was also used in [41]. This paper considered the control of a small-scale model of the continuous casting process. In continuous casting, the quality of the casted metal depends to a considerable degree on the flow patterns in the mold. These flow patterns are shown in Figure 1a. The desirable flow pattern is a double-roll flow pattern, while a single-roll flow pattern should be avoided. The flow inside the mold was measured using Ultrasonic Doppler Velocimetry (UDV) sensors. UDV sensors measure the velocity field inside the volume of the mold. Because of this ability to look inside, UDV sensors can be classified as a specific tomographic modality or at least as sensors with properties similar to tomographic sensors [42,43]. It can be seen from Figure 1a that the flow pattern is determined mainly by the initial direction of the flow from the nozzle. This initial part of this flow can be approximated by a line with varying angles (see Figure 1b). This angle should be kept within acceptable limits by a controller and electromagnetic actuator.

What is important from the viewpoint of sensor data processing is the fact that this is a problem with very simple geometry. The analysis of the data can be reduced to finding the angle of the jet flow exiting from the nozzle. Further research described in [44] has shown that the shape of the exiting jet flow has a rather banana-like than straight-line shape, and a third-order polynomial better parametrizes the whole problem than just a linear function. However, this is merely a development of the same idea, just with slightly more complex geometry.

Another method enabling the use of tomography measurements without performing blind pixel-based image reconstruction is state estimation. This approach makes use of the techniques that were developed for finite-dimensional state-space system models. The original developments (Luenberger observer, Kalman filter) were for linear systems. Later, they were extended to nonlinear systems (extended Kalman filter and related techniques). State estimation techniques are a standard part of control theory described in many textbooks. A detailed treatment can be found, e.g., in [45].

The use of state estimation techniques for the reconstruction of tomography data is based on a fairly simple idea. A tomographic sensor is connected with a process whose PDE description is converted into a finite-dimensional state-space model using some of the methods mentioned above. This state-space model is usually also time-discretized. It describes the dynamics of the process. In papers focused on tomographic sensors, this fact is often stated by saying that it describes the evolution of the process. Mathematically, this is a set of ordinary differential or difference equations.

A tomographic sensor produces measurements that depend on the internal states of the dynamic model and represent system outputs. This relationship is expressed by the so-called output equation of the state space model. Considering the time-discretized variant of state-space Equation (1) we have the equations in the following form

(6) $$\begin{array}{c} x(k+1)=Mx(k)+Nu(k)+\xi \left(k\right)\\ y(k)=Cx(k)+Du(k)+\eta (k) \end{array}$$

This is the linear time-invariant case. These equations may be generalized into more general cases. Matrices of the model become time-dependent if the system is time-varying. If the system is nonlinear and time-varying, the equations can be written as

(7) $$\begin{array}{c} x(k+1)=f\left(x(k),u(k),\xi \left(k\right),k\right)\\ y(k)=g\left(x(k),u(k),\eta (k),\;k\right) \end{array}$$

In these equations, ***x*** stands for a vector of internal state variables (e.g., concentration distributions), and ***y*** is the vector of the measured outputs. Vectors ***ξ*** and ***η*** are noises: process noise and measurement noise, respectively. These vectors are not included in the continuous version of the state space Equation (1). They are added to the discretized version because these noises are the standard way the model uncertainties and measurement errors are considered in Kalman filtering and LQ control [45]. System input (manipulated variables, disturbances) is denoted with ***u*** and *k* stands for the discrete time.

The task of a state estimator is generally to estimate the values of state variables from available output measurements. In this specific application, the measured outputs are tomography measurements. The use of state estimation enables us to find the values of internal states (i.e., the variables to be sensed by tomography) without performing pixel-based image reconstruction. This approach to tomography data analysis is particularly useful in connection with control approaches based on state-space models.

**Figure 2:**
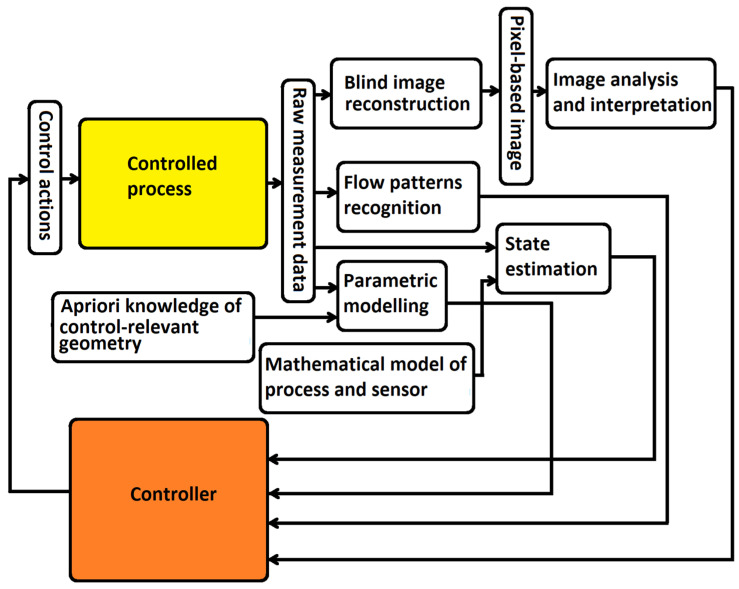
Main options for closing the control loop using tomographic measurements.

The purpose of this section was not to give a detailed exposition of the issues associated with tomography data processing. Such a presentation would require another complete review paper. This section’s main objective was to illustrate that the connection between sensor and controller in tomography-based control is much more complicated, and more options exist than in control tasks with standard sensors. The various options for connecting a tomographic sensor into a control loop outlined in this section are summarized in Figure 2. It shows that besides the “long feedback loop” going through image reconstruction and reconstructed image analysis, we have several shorter options that are generally more suitable for real-time control.

## 3. Control Techniques, Methods, and Applications

### 3.1. Control Based on Distributed Models

As has already been stated, tomographic sensors enable distributed sensing. As a result, it is pretty natural to combine them with control approaches that exploit the distributed parameters nature of the controlled process. For this reason, we will start this treatment with this class of approaches. A very important category of processes with distributed parameters is the processes that include single-phase or multiphase flows. Applications of tomography-based control to flow processes have been reported since the very beginnings of industrial tomography.

#### 3.1.1. Concentration Distribution Control

**Figure 3:**
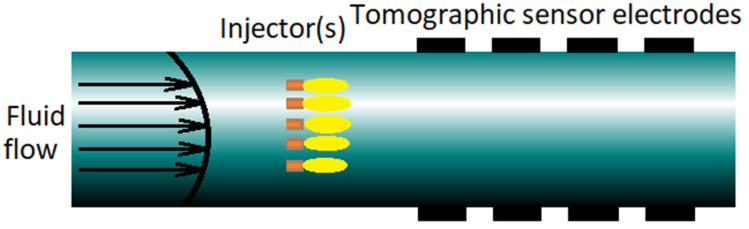
Schematic sketch of the concentration distribution control process.

A very basic control task related to flow control is the control of the concentration distribution of a substance in a fluid flow along a straight pipe with a circular cross-section. The concentration profile is controlled by injecting strong concentrate into the flow through one or more injectors. Their flow rates are considered manipulated variables. The control action is based on concentration measurements performed by an electrical impedance tomography system located downstream from the injection point. The structure of this process is shown in Figure 3.

From one viewpoint, this is a relatively simple, specific process. However, as far as the structure of its mathematical model is concerned, this process reflects the general nature of many fluid flow processes, including liquid/liquid and solid/liquid mixing. Thus, it can be viewed as a paradigmatic controlled process for tomography-based control. For this reason, its control was addressed in many papers, most notably in several papers and a PhD thesis written mainly by S. Duncan, A. Kaasinen, and their co-authors [46,47,48,49,50,51]. Therefore, we will describe this process in some detail. A mathematical model of this process has been given several times in slightly different variants in the papers cited above. The following equations are based mainly on [51].

The concentration of the injected substance can be described by a PDE model—convection–diffusion equation

(8) $$\frac{\partial c\left(\xi ,t\right)}{\partial t}=-v\left(\xi \right)\times \nabla c\left(\xi ,t\right)+\nabla \times \kappa \left(\xi \right)\nabla c\left(\xi ,t\right)+q\left(\xi ,t\right)$$

where ***ξ*** is the vector of space coordinates within the domain of interest Ω (finite segment of a pipe). This domain is generally three-dimensional, but it can be two-dimensional if it is a straight pipe with circular symmetry. The concentration distribution is denoted as *c*(***ξ***, *t*) where ***ξ*** ∈ Ω and *t* ≥ 0, ***v***(***ξ***) is the velocity field and *κ*(***ξ***) is the diffusion coefficient. The source term ***q***(***x***, *t*) describes the injection of substance B into the flow of substance A. If there are multiple injectors, this term can be expressed as

(9) $$q\left(\xi ,t\right)=\Lambda u\left(t\right)=\Lambda \left[\begin{array}{ccc} u_{1}\left(t\right) & \cdots & u_{K}\left(t\right) \end{array}\right]$$

where ***u***(*t*) is the vector of manipulated variables and **Λ** is a linear map that depends on the geometry of the injection and the number of injectors *K*.

Initial and boundary conditions can be specified as follows

(10) $$c\left(\xi ,0\right)=c_{0}\left(\xi \right),\;\xi \in \Omega$$

(11) $$c\left(\xi ,t\right)=c_{in}\left(\xi ,t\right),\;\xi \in \Omega_{in}$$

(12) $$\frac{\partial c\left(\xi ,t\right)}{\partial n}=0,\;\xi \in \Omega_{wall}\cup \;\Omega_{out}$$

Here *c_in_*(*t*) is the time-varying concentration at the input boundary, i.e., it is generally assumed that even the fluid entering the pipe can contain a fraction of the substance injected by the injectors. This variable is a disturbance from the viewpoint of feedback control. 

PDE model is further space-discretized to obtain a finite-dimensional model. This can be done using FEM (e.g., [47,48,49,50,51,52]) or finite difference approximation in the direction of the pipe centerline [49,50]. Regardless of the method used, the result is a high order state-space model that can be written in a time-discretized form as

(13) $$c\left(k+1\right)=Ac\left(k\right)+B_{1}u\left(k\right)+B_{2}d\left(k\right)+w\left(k\right)$$

where ***c***(*k*) is the space and time-discretized concentration, which plays the role of a state vector, ***u***(*k*) is the vector of manipulated variables defined in Equation (9), disturbance ***d***(*k*) is input concentration defined in Equation (10), and ***w***(*k*) is a stochastic process representing modeling uncertainties, random components of input signals and other stochastic influences. Assuming that the velocity field and the diffusion coefficient in Equation (8) are not time-varying, the matrices in Equation (13) are constant, i.e., they describe a linear time-invariant finite-dimensional system. It can be used instead of the PDE model (8) in control design. That means that this approach is a sort of early lumping design.

Concentrations are measured using electrical impedance tomography. Depending on the properties of the fluids, either conductivity or permittivity measurements (i.e., electrical resistance or capacitance tomography) are used. Conductivity measurements (i.e., a conductive fluid consisting of components with different conductivities) will be assumed here. However, the main principles would be the same if permittivity measurements were considered.

Electrical tomography does not provide simple and direct information about concentrations. The available measurements are voltages between different pairs of electrodes produced by varying current injection patterns (*j* = 1, …, *N*_I_) applied to the electrodes. These voltages depend on the conductivity distribution *σ* within the pipe, and this conductivity distribution depends on the concentration of injected substance *c*. Voltage measurements for one current injection pattern can be expressed by a compound formula

(14) $$V_{j}=U_{j}\left(\sigma \left(c\right)\right)+v_{j}=U_{j}^{*}\left(c\right)+v_{j}$$

where ***V****_j_* are the voltage measurements, and ***U****_j_* maps the conductivity to the measured voltages. Vector ***v****_j_* represents additive measurement noise. If the controlled process is slow enough, the conductivities can be considered constant during the whole frame, where the full set of all current injection patterns is used. In this case, all measurements and conductivity mappings can be combined into one vector and one mapping [52]. Equation (9) can then be written as

(15) $$V\left(k\right)=U\left(\sigma \left(c\left(k\right)\right)\right)+v\left(k\right)=U^{*}\left(c\left(k\right)\right)+v\left(k\right)$$

All variables have the same discrete time, i.e., the time necessary for performing a full set of current injections must be significantly shorter than the controller sampling period. However, these stationarity assumptions are often not satisfied in the real-time control. This non-stationarity can be handled in several ways. It is possible to use a substantially reduced set of current injection patterns or even one pattern optimized in such a way that it provides as much information as possible [53]. The observation equation has then the same form as Equation (15), but it uses just one measurement or a small number of measurements. As a result of this, the concentrations must be computed on the basis of a smaller amount of data. Alternatively, this observation equation can be considered time-varying where the mappings are different each discrete time *k* depending on which current injection pattern *j* is used.

Equation (15) can be kept in its nonlinear form, or it can be linearized and written in deviation variables around an appropriately selected operating point

(16) $$V\left(k\right)=U_{0}+J\left(c\left(k\right)-c_{0}\right)+v\left(k\right)\Rightarrow \;\Delta V\left(k\right)=J\Delta c\left(k\right)+v\left(k\right)$$

where ***J*** is the Jacobian of the function U*(***c***(*k*)) evaluated at the operating point *c*_0_.

Equations (13) and (16) or (15) are then of the same form as Equation (6) or (7). They can be used to design a state estimator using either a linear version of the Kalman filter or some of its nonlinear versions, e.g., an extended Kalman filter. State estimation was originally proposed as an approach to solving the inverse problem and obtaining the concentration (or other) data from voltage measurements where the dynamic Equation (13) was used in order to bring additional information and make the whole procedure feasible, especially in the non-stationary cases, where observation Equation (15) includes just a limited amount of data.

This means the original motivation was mainly data reconstruction. However, this setting is very suitable for control. Equations (13) and (15) or (16) provide a good starting point for control methods based on state-space models. It is just necessary to consider that although observation Equations (15) and (16) can be regarded as output equations of a state-space model, they provide measured outputs that are not controlled variables. The controlled variables are concentrations at a selected region. Most naturally, the output boundary can be considered. This can be described by an additional output equation

(17) y(k)=Cc(k)

Matrix ***C*** can be zeros and ones matrix that selects just the concentrations in the region of interest as controlled outputs. This form of output equation can also cover other cases of control objectives, e.g., an average concentration in a chosen region of interest can be the output variable.

We described this concentration control process in some detail because it corresponds well with the fundamental characteristics of tomographic sensors. Corresponding to the distributed sensing achievable by tomography, there is a distributed parameter model of the process. This model is handled using the early lumping approach, i.e., it is converted into a high order state-space model, which can then be used as a starting point for several controller design approaches.

Early papers by S. Duncan [49,50] used PI controller. This controller is suitable for SISO systems. In accordance with this, only one injector was used, and the average concentration at a particular pipe point was the controlled variable. The control was tested in simulation only. Control performance was rather poor, marked by oscillatory responses to set-point changes. These oscillations were caused by the significant delay because of the flow from the injector to the measuring point. It is well known from the theory of time-delay systems that these oscillations can be eliminated either by decreasing the proportional gain or by adding the Smith predictor structure to the PI controller [54]. However, neither of these approaches was used in [28,29].

Since a state-space model is available, it is possible to use advanced model-based controllers instead of PI control. This was done in several papers [46,47,48,51]. The control approach of choice was the Linear Quadratic (LQ) controller. This controller is based on quadratically optimal state feedback in the form

(18) u(k)=−K(k)x(k)

where ***x***(*k*) is a state vector, i.e., it is the vector of concentrations ***c***(*k*) if model (8) is the controlled plant. ***K***(*k*) is an optimal feedback gain matrix calculated using a discrete-time matrix Riccati equation [55]. Equations (13), (16), and (17) provide a standard setting for the design and straightforward application of LQ controller. It was only necessary to take into account that the LQ controller in its basic form is a regulator, i.e., its objective is to drive states and system output to zero. In this case, it is not an appropriate objective because the control objective is to obtain a specified nonzero reference concentration ***y***_ref_ at the output boundary. For this reason, the structure of the controller had to be changed to

(19) $$u\left(k\right)=\bar{u}-K\left(k\right)\left(c\left(k\right)-\bar{c}\right)$$

where $\bar{u}$ and $\bar{c}$ are steady-state values corresponding to the state, where the reference concentration ***y***_ref_ is achieved. This modification introduces feedforward action into the controller. Set-point enters the controller indirectly as a variable on the basis of which the steady-state values are computed. The controller described by Equation (19) is used by all of the papers [46,47,48,51]. The state vector (concentrations), which is input to the controller (19), is estimated either using a Kalman filter based on a linearized observation equation [46] or using an extended Kalman filter based on a nonlinear observation equation [51].

This control application of tomography may seem to be a fairly nice example of the early lumping approach applied to a control of a distributed parameter system connected with distributed sensing. It includes a well-established mathematical model as well as an advanced control method. Unfortunately, this concept of tomography-based control may also be a blind alley. The controlled process considered here is relatively simple, and it was the subject of several papers published over more than one decade. Nevertheless, this concept has never gone beyond simulation testing. None of the papers gives more than simulated responses. Moreover, it also seems that this research line found only a very limited continuation in the last decade.

#### 3.1.2. Microwave Drying Process

Looking to the development following 2013 when [51] was published, almost no papers follow this concept and attempt to transfer it into other controlled processes. The most notable exception is the paper by Hosseini et al. [56] published in 2020. This paper is focused on the control of a microwave drying process. In microwave drying, a porous dielectric material with high moisture content is placed inside a cavity and exposed to microwave sources (magnetrons). In paper [56], the control objective is specified in the following way: “The objective is to reach as homogeneous moisture distribution as possible inside the porous material and the average moisture of the material should follow the desired moisture level.” Power levels of microwave sources are used as manipulated variables. The output moisture is measured using electrical capacitance tomography, i.e., using a sensor whose behavior is similar to the sensor described by Equations (14) to (16) but with permittivity instead of conductivity.

Industrial tomography is the key enabling technology for feedback control of this process because it is the only technology that can measure the moisture distribution inside the material. Alternative sensor technologies can provide only surface-related information which is not enough for efficient control. For this reason, paper [56] is also likely to be the first paper to treat the question of feedback control of microwave drying. It should also be noted that efficient feedback control would be a very important innovation in the case of microwave drying because if the control of microwave sources is not based on the measurements taken inside the material, there is a danger of hot-spot formation [57]. This danger is particularly high in the case of drying porous polymer foams, where it can lead not only to low-quality processing but also to foam ignition danger [58].

The control design concept used in [56] is similar to the concept described in the preceding section for the concentration distribution control process. The microwave drying process was modeled using a parabolic PDE model. This model was space discretized; the LQ controller was designed and tested in simulation. Since the control objective is to reach a nonzero set-point, a modified version of LQ control similar to the controller (19) had to be used. The simulation results presented in [56] are promising. However, this paper does not go beyond simulation, and no follow-up papers going further to implementation have been published.

#### 3.1.3. Inline Fluid Separation Process

**Figure 4:**
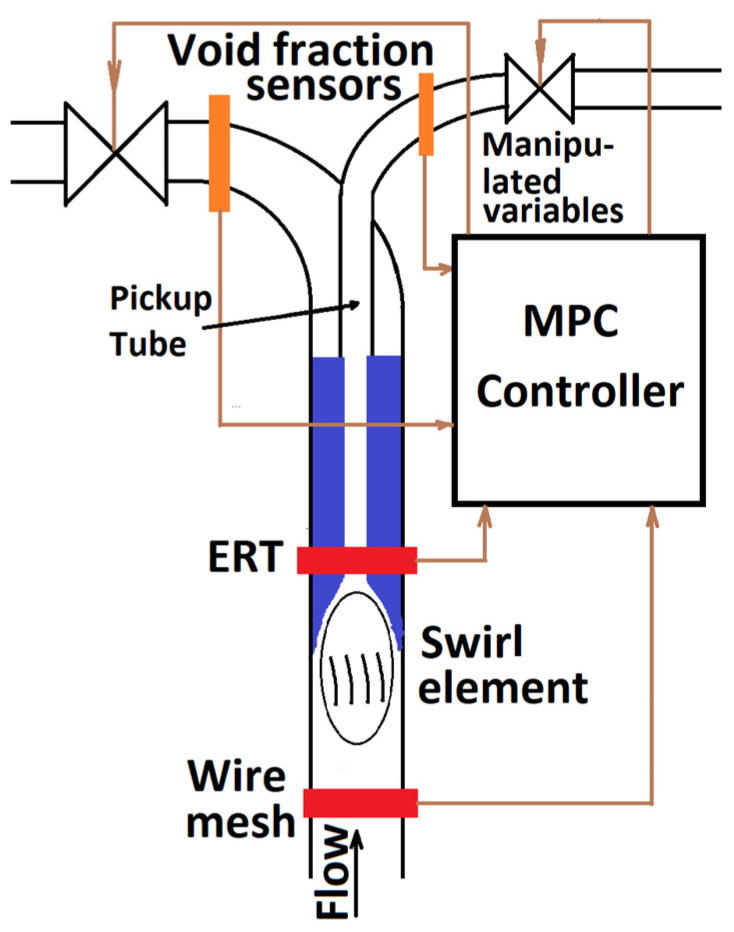
Schematic sketch of the swirled separator process.

A similar controller design methodology based on the concept of space-discretized PDE models was also applied to control of gas–liquid inline swirl separator. This control design was described in recent (2020) papers [59,60]. The structure of the gas–liquid inline swirl separator process is outlined in Figure 4. The central component of this process is a vaned swirl element generating the swirling flow. This swirling motion should generate a core of the lighter phase, which is then captured by the central pickup tube, while the heavier phase should be captured by the outer tube. In order to improve this phase separation, the flows through the tubes are controlled by two valves. These valves are used as manipulated variables.

Conventional control of this process is explained in a recent paper [61]. It is inherently indirect and based on the so-called pressure drop ratio, i.e., the ratio of the pressure differences between the pressure in the inlet tube and the two outlet tubes. It is rightly stated in [61] that such an indirect control approach can reduce the efficiency of these devices significantly and result in violations of the environmental requirements. The authors also propose some extensions and improvements to the control based on the pressure drop ratio. However, these extensions are just variations of the same basic structure, e.g., they add feedforward action or make the setpoint of the ratio controller variable. On the contrary, the structure shown in Figure 4 allows significantly more direct control because it includes several tomographic sensors.

Void fraction sensors in the outlet tubes can be either ERT or wire-mesh sensors, and they measure cross-sectional volumetric gas fractions in the outlet flows. From the viewpoint of the control system, all these variables can be used as controlled variables. The ERT sensor monitors the gas core created by the swirl element. This measurement can be used both as a controlled variable and as a measured disturbance. The wire mesh sensor in the bottom measures the gas fraction and gas velocity of the incoming flow. In this way, it provides some kind of advanced information for the controller. It can be used as a measured disturbance for controller feedforward action.

The underlying concept used by paper [59] is the same as was described in the previous sections on microwave drying and concentration distribution control. The starting point is a simplified PDE model of the flows inside the separator process. This model is space discretized using the finite volume method. The resulting finite-dimensional model is then linearized. The result of this procedure is a high-order state-space model of the form (1). Unlike previous sections, this paper uses not LQ but MPC approach for controller design. However, this MPC controller is developed in a really minimalistic form. Although the paper introduces the control structure shown in Figure 4, this structure is rather an ideal concept that should be achieved in future development. The really developed MPC controller is much more modest. It does not use the information from the inlet wire-mesh sensor. Rather, the ERT sensor measurements serve as a measured disturbance, while the measurements from the void fraction sensors are controlled variables. The controller is unconstrained, and its cost function considers only squared differences between measured void fractions and perfect separation conditions, i.e., increments of manipulated variables are not included in the cost function.

Although the controller was tested in simulations, these simulation tests are still at a very early stage. At this moment, it can be considered a very interesting concept. However, only future development can show its real strength. The open question is not so much the use of MPC but whether the space-discretized PDE model is really the most suitable option. Although [59] uses this version of the model, in [61] the authors consider using either identified black box/grey box models or neural network models.

#### 3.1.4. Issues with Control Based on Early-Lumped PDE Models

Papers [56,57,58,59] are the only papers using the concept of early lumped PDE models for control with tomographic sensors published after 2013. Trying to analyze the reasons for this somewhat discouraging situation, at least two principal issues can be identified.

The first issue to consider is the use of linear quadratic control. This control method was originally developed for aerospace applications where modeling the uncertainties by white or colored noise disturbances such as in Equations (6) and (7) is more or less adequate. However, it is generally known that this is not an adequate model for industrial process control and that the application of the LQ approach to industrial control problems is not really a success story [62]. Moreover, the version of set-point tracking described by controller (14) is sensitive to model inaccuracy. If the steady-state values are calculated using an inaccurate model, there is no way to compensate for this, and steady-state control error appears. A more robust version of linear quadratic set-point tracking can be obtained by introducing the integral of control error to the controller [55], but this has never been used in the context described above.

Generally speaking, the importance of linear quadratic control is mainly historical. LQ control was the first control method based on quadratic optimality criterion and state-space models, but it has never found significant industrial acceptance. On the other hand, model predictive control (MPC) [63] is related to LQ control in many important respects. It also usually uses a quadratic criterion of optimality, and it is based on a model of the process. LQ control is sometimes referred to as the zeroth generation MPC because of these similarities. However, despite these similarities, MPC is much more suitable for industrial use than LQ control. In particular, MPC features a unique ability to handle constraints, a wide range of acceptable models (state-space models, step-response models but also e.g., fuzzy models), and suitability for control of multivariable processes. Nonzero set-point tracking can also be achieved in a straightforward way, and no awkward modifications such as in the case of LQ control are necessary. For these reasons, MPC has found wide industrial acceptance. Nowadays, it is the standard method of choice for control tasks where PID control is unable to achieve desired control performance.

Although the idea to apply MPC in a context similar to the application of linear quadratic control described above may seem obvious, there are just two attempts in this direction. One attempt done in [59] has already been described, and it is rather a concept than a fully-fledged MPC application. The second attempt is the paper by Sbarbaro and Vergara [64] published in 2015. This paper uses electrical impedance tomography modeled by Equations (14) and (15) while the controlled plant is a generic discrete-time state-space model with a linear dynamic equation and nonlinear output (observation) equation

(20) $$\begin{array}{c} x(k+1)=Mx(k)+Nu(k)\\ y(k)=g\left(x(k)\right) \end{array}$$

This structure corresponds well with the model of the concentration distribution control process described by Equations (18) and (20) as well as with the space-discretized model of the microwave drying process. Similar correspondence could be found with other processes where tomographic sensors can be applied. That means the MPC design proposed there could be fairly general. However, the MPC design in [64] does not go too far. The paper includes just standard textbook equations of analytical unconstrained MPC controller combined with a nonlinear observer. The resulting control design is tested using a simple numerical example.

The second issue is the dimensionality of the model. PDE models are space-discretized using the finite element method (FEM) or finite differences method in the above-mentioned papers. This is an appropriate approach for numerical simulation, but it does not provide good control-oriented models. For instance, the order of the state-space model used in [56] is 3670. This extremely high order is likely to result in high computational demands and ill-conditioned computations. Since the processes considered are described by parabolic PDE, it might be better to use the well-known fact that parabolic systems have a spectral gap between finite-dimensional slow and infinite-dimensional stable, fast modes [65]. This enables an accurate approximation of such PDEs using relatively low-order models based on slow modes. Such models can be derived using spectral methods or the Karhunen–Loève method. For instance, paper [66] applies these methods to distributed parameters processes with convection-diffusion phenomena and compares the quality of resulting low order models. In this paper, an acceptable approximation is always achieved with model order less than ten, which is by order of several magnitudes less than the order of models resulting from FEM.

Using low order models derived from parabolic PDE models on the basis of spectral methods or the Karhunen–Loève method is an open research direction for further development of tomography-based control. The use of these methods and their advantages are not as straightforward as they might seem at first sight. All approximation methods can be interpreted in such a way that the spatiotemporal PDE variable *y*(*x*, *t*) is expanded by a set of spatial basis functions Φ*_i_*(*x*)

(21) $$y\left(x,t\right)=\overset{\infty }{\underset{i=0}{\sum }}\Phi_{i}\left(x\right)y_{i}\left(t\right)≅\overset{n}{\underset{i=0}{\sum }}\Phi_{i}\left(x\right)y_{i}\left(t\right)$$

where the infinite upper limit is replaced by a sufficiently high finite *n*. The low order of the approximated model in spectral methods can be achieved because the spatial basis functions are global, i.e., they are nonzero in the whole domain of interest. On the other hand, FEM can be understood as a method with local spatial basis functions (nonzero only within one element). As a result of this, high *n* is necessary for a good approximation.

However, this high *n* simplifies the form of the output equation. If the model output has to correspond with tomography measurements, (i.e., with measurements distributed within some subdomain such as, e.g., the output boundary), it is enough to take the values of the elements located in the subdomain of interest, if FEM is used. We obtain a simple output equation such as Equation (17) where ***C*** is zeros and ones matrix. On the contrary, the construction of the output equation becomes more complicated if any method with global spatial basis functions is used. Despite this problem, methods with global spatial basis functions are worthy of research attention because control designs based on models with thousands of state variables are unlikely to find industrial implementation.

### 3.2. Control Based on Lumped Parameters Dynamical Models with a Static Model of Distributed Variables

**Figure 5:**
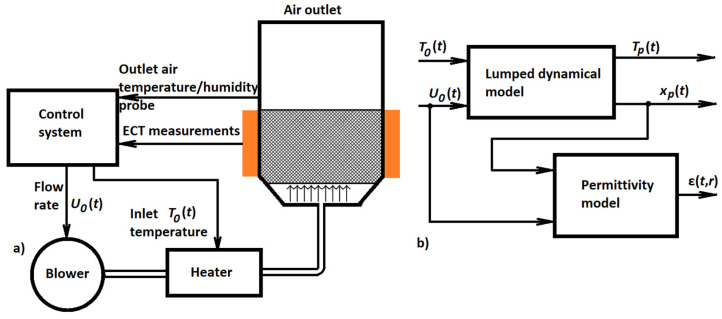
(**a**) Schematic sketch of the fluidized bed dryer and its instrumentation (**b**) structure of its control-oriented mathematical model.

Although no published paper has ever used spectral and related approximation approaches in connection with tomography, several papers use methods that are different in concept but similar in their results. These are the papers by Villegas, Duncan, Wang, and others focused on the control of batch fluidized bed dryers [67,68,69,70]. A schematic sketch of this dryer and its instrumentation is shown in Figure 5a.

Such dryers are used in a wide range of industries (food, pharmaceutical, chemical). In these dryers, solid particles are transformed into a fluid-like state by forcing a gas (typically air) to flow in an upward direction through the bed. One of their main advantages is that the high turbulence arising in the bed provides high heat and mass transfer, as well as a high degree of mixing of the solids and gases within the bed. However, this turbulence may also result in non-uniform distribution of the solid phase (powder) inside the bed and local variations of moisture and other physical properties of the powder. This is the motivation for introducing a tomographic sensor that can observe these local variations inside the dryer.

There is actually a significant degree of similarity between this process and the concentration distribution control process shown in Figure 3. Both of them are fluid flow processes. Electrical tomography is used to measure a distributed controlled variable. However, the modeling approach is different. The authors of [69] use a dynamic model built from the very beginning as a simplified lumped parameters model based on mass and energy balances of the solid, gas, and bubble phases. Its input variables are inlet air temperature (*T*_0_) and velocity (*U*_0_), while output variables are particle moisture (*x*_p_) and temperature (*T_p_*). Although there are two input variables, it was shown by a sensitivity analysis published in [46] that the inlet air velocity has a much more significant influence on the performance of a fluidized bed dryer than the inlet air temperature. For this reason, it is enough to use this velocity as the only manipulated variable while the inlet air temperature can be kept constant.

The distributed model describes the variation of permittivity and thereby also the distribution of the particle moisture over the cross-section of the dryer. It is described by an algebraic expression

(22) $$\varepsilon \left(r,U_{0},x_{p}\right)=f_{R}\left(U_{0},x_{p}\right)+\overset{N}{\underset{i=0}{\sum }}c_{i}\left(U_{0},x_{p}\right)J_{0}\left(r\frac{\lambda_{i}}{R}\right)$$

where *R* is the radius of the dryer, *r ∈* <0,*R*>, *f_R_* and *c_i_* are real functions and *J*_0_ is the Bessel function of the order zero, and *λ_i_* the *i*-th positive zero of this function. The structure of the mathematical model combining lumped dynamical model and (22) is shown in Figure 5b.

The choice of Bessel functions in the permittivity distribution model (22) is motivated by their suitability for cylindrical coordinates. Model (22) is not a first-principles model but a suitable model structure whose components are to be obtained by identification from experimental data. This was done in [69] using the data from a small laboratory scale dryer Sherwood M501. Good approximation was obtained with *N* = 4 and bivariate polynomials up to second order in the positions of *f_R_* and *c_i_*.

It should be noted that if the modeling started from a PDE model and some method with global spatial basis functions were used for space discretization, there would be similarities between the resulting model structure and the structure shown in Figure 5b. Again, it would be a relatively low order lumped parameter dynamical model with a static output function. This output function could be linear if it directly described the output variable of interest. However, it would be nonlinear similarly to model (22) if it described variables such as permittivity or conductivity. This is because of the nonlinearity of the relationship between variables such as concentration or moisture on the one hand and conductivity or permittivity on the other hand.

The control objective was to achieve the desired moisture distribution over the cross-section of the dryer. This desired moisture distribution can be converted into a corresponding desired permittivity shape as expressed by the summation term in model (22). The authors of [69,70] used offline optimization. They calculated a set of optimal values of *U*_0_ corresponding to a set of particle moisture *x_p_* values for a specified desired permittivity shape and identified coefficients in model (22). The relationship between *x*_p_ and optimal *U*_0 *opt*_ resulting from this optimization was then approximated by a third-order polynomial

(23) $$U_{0\;opt}=p\left(x_{p}\right)=a_{3}x_{p}^{3}+a_{2}x_{p}^{2}+a_{1}x_{p}+a_{0}$$

**Figure 6:**
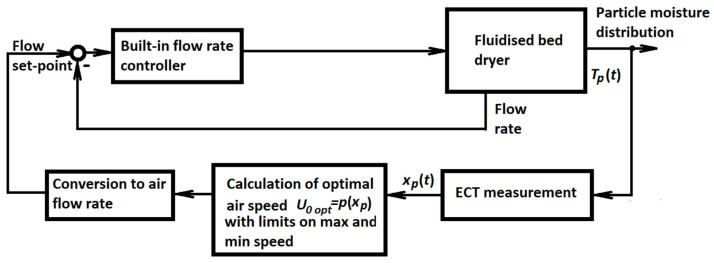
Schematic structure of the fluidized bed dryer control system.

Putting together the descriptions somewhat scattered between [44] and [45], the resulting control structure can be visualized using the scheme in Figure 6.

The fluidized bed dryer used for experiments was a laboratory-scale dryer Model 501 produced by Sherwood Scientific. Technical details of this dryer are given by the manufacturer in [71]. This device incorporates an air pump, heating coil, and temperature measurement (with control and timer) in its basic configuration. Microprocessor control of airflow, inlet air temperature, and drying period is available. The device configuration used in the experiments included an optional moisture probe, and it was further equipped with an ECT system with a data acquisition rate of 120 frames per second [69]. This instrumentation corresponds to Figure 5a.

A standard humidity probe measures just the humidity of the outlet air and not the moisture content in the particles being dried. These particles are scattered over the whole cross-section of the dryer. As a result, their moisture content cannot be measured by a single-point probe, but electrical tomography must be used instead. On-line evaluation of the average particle moisture is based on ECT images reconstructed using Landweber iteration [72]. Therefore, this control is basically a simple cascade control loop with a single controlled variable in the master loop. However, the tomography sensor evaluating the whole cross-section of the dryer is necessary to obtain the value of this single controlled variable.

The master controller in the structure shown in Figure 6 and described by polynomial (23) is a purely static term. It can be understood as a nonlinear version of the proportional controller. The airspeed, which is the manipulated variable of this controller, must be limited. In particular, its lower bound is important to ensure that the speed will never fall below minimum fluidization velocity.

The control objective is to achieve an appropriate moisture distribution, which is translated into achieving the corresponding reference permittivity shape. It is important to note that there is no reference input to the controller, but the control objective is expressed indirectly by the values of coefficients in approximation polynomial (23). If the desired shape changes, repeated off-line optimization must be performed, and the values of coefficients in polynomial (23) must be updated accordingly.

Regarding the relationship between the desired and real permittivity shape, the controller can be classified as a feedforward controller whose robustness is inherently limited. If there is any change in the behavior of the dryer, the functions in the permittivity distribution model (23) should be updated. However, there is no way for the controller to learn about this and a mismatch between desired and real permittivity shapes can appear. It is evident that there are many ways to improve the control. The availability of lumped dynamical model and electrical tomography sensor and other instrumentation shown in Figure 5 gives many opportunities for control improvement. However, this is an open research direction. At this moment, it seems that the research line documented in [67,68,69,70,71] has not found further continuation, at least as far as the modeling and control approaches are considered.

### 3.3. Experimental Approaches: Identified Models, Empirical Controller Tuning

#### 3.3.1. Control of a Wurster Fluidized Bed

However, a continuation of development can be found if the nature of the controlled process is considered, such as in a recent (2020) paper [73] by H. Wang and others. There is also a personal continuity because H. Wang was one of the authors of the papers on the control of fluidized bed drying cited above [67,68,69,70]. The process considered by Wang is similar in many respects to the fluid bed dryer. It is the so-called Wurster fluidized bed, commonly used for coating pellets in the pharmaceutical industry. This process has a concentric cylindrical tube (Wurster tube) inside a conical chamber. This internal structure results in two flow regions: the coating region (inside the Wurster tube) and the annular region (outside the tube). In this process, a coating solution is continuously sprayed into the fluidized bed. Pellets are covered with the solution and dried with hot air. Complex flow behaviors inside this fluidized bed cannot be adequately captured by the local flow information provided by conventional point-based measurements. For this reason, any real-time control and optimization are inherently difficult, and the operation of the Wurster fluid bed coating process involves a significant deal of trial and error.

This motivates the use of electrical capacitance tomography. Similar to the dryer described above, the ECT images are reconstructed using Landweber iteration. These images are then processed to obtain information about the flow regime. Several regimes can exist: bubbling, intermittent, and plug flow. It is important to keep a minimum fluidization state and avoid intermittent flow, plug flow, and defluidization. Images reconstructed from ECT measurements are processed to obtain the gas volume fraction in the annular region and its cycle time (measured from the number of times this signal crosses its average value). The values of these parameters are specific for different flow regimes, and the undesirable regimes can be avoided by keeping gas volume fraction and cycle time at specified set points.

This objective was achieved in [73] by two uncoordinated control loops. PID controller controlled the gas volume fraction in the annular region using the fluidization air rate as a manipulated variable. Another control loop used an on-off controller controlling the cycle time using the pump for spraying coating solutions as an actuator. On-off controllers do not require any tuning parameters except for hysteresis. PID controller was tuned experimentally using the Ziegler–Nichols method [74]. This control structure has been found to be effective for this coating process by the authors. Nevertheless, it is evident that an advanced tomographic sensor with a sophisticated data analysis algorithm was connected with a very basic control system.

It is clear that even if this controlled process is quite similar to the fluidized bed dryer described above, the control design approach is significantly different. Neither distributed nor lumped dynamical model was used for controller design and tuning. A very standard PID controller was tuned using old empirical Ziegler–Nichols rules. Tomography measurements were eventually condensed into one scalar value obtained on the basis of a reconstructed image that could be used as a controlled variable of the PID control loop.

A similar approach, i.e., identification-based modeling or empirical controller tuning, is fairly common. We have already seen that this was a considered option in the case of the swirled separator process. Similarly, the model of the distributed permittivity (22) in the case of the fluidized bed dryer was also obtained by measurements of the real process.

#### 3.3.2. Control of Microwave Drying

Furthermore, we have mentioned the microwave drying process. In [56] it is modeled by the PDE model and feedback-controlled using an LQ controller. Further development of this research by the same authors can be found in [75]. The conveyor belt microwave drying process is identified as a state-space model of the order eight with input delays. It can be described by the following expression:

(24) $$\begin{array}{c} x(k+1)=Ax(k)+B_{1}u_{1}(k-T_{d1})+B_{2}u_{2}(k-T_{d2})\\ y(k)=Cx(k) \end{array}$$

where *u*_1_ is the power applied to magnetrons, *u*_2_ is the average input moisture, and *y* is the average permittivity change measured by the ECT sensor corresponding to the change of average output moisture. The input moisture was calculated on the basis of weighting the sheets of wet foams on a digital scale and the known weight of dry foam sheets.

Model structure (24) is relatively simple. Average moisture values are used, and power levels of all magnetrons are changed in the same way, i.e., the power level can be considered as a single manipulated variable. This approach can be extended into a more general case, and model (24) can be turned into a multi-input multi-output (MIMO) system. Paper [75] is focused on identification only. It does not cover controller design. However, it is more than evident that the 8th order model (24) is a much better starting point for controller design than the model with order 3670 described in [56]. Input moisture, which is the second input to model (24) is a disturbance for the controller. Paper [58] describes a microwave tomography sensor intended to be used for online measuring of this moisture. In this way, all building blocks for a controller based on an identified model with measured disturbance feedforward are prepared in the case of the microwave drying process. The authors of [75] performed the first experiments with implementation and experimental testing of the controller based on identified model (24) using PID and LQ control approaches [76]. Although there is space for further development, the controllers are at least implementable and able to achieve their control objectives, which would be impossible using the controllers-based high order models described in [56].

#### 3.3.3. Control of Continuous Casting of Metals

**Figure 7:**
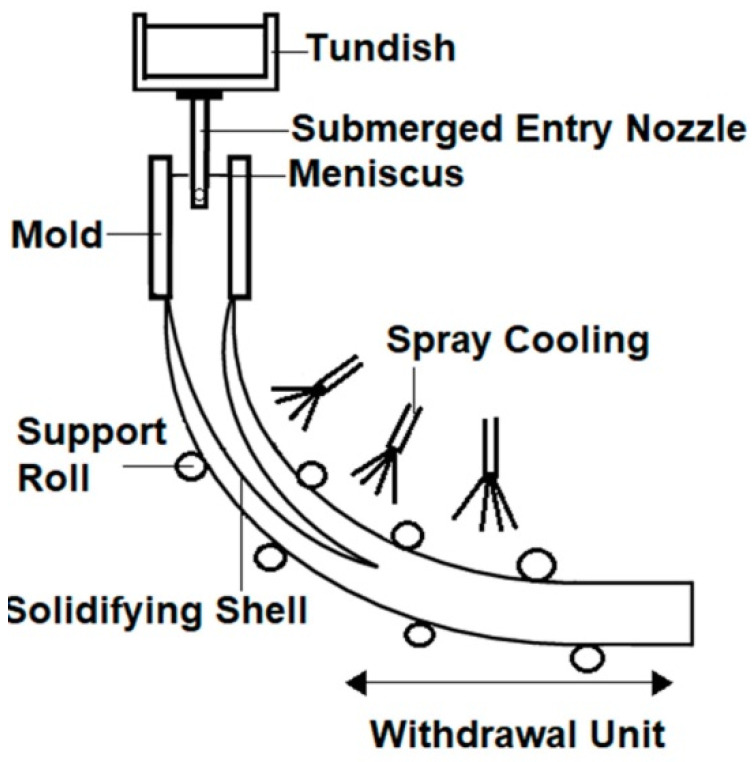
Schematic of the continuous casting process.

Another important process for which tomography-based control was proposed is the continuous casting process. Continuous casting is very widely used for both ferrous and non-ferrous metals. Its principle is as follows: molten metal from furnaces is transported to the top of the casting machine using a large vessel called a ladle, and from this ladle, it is poured into a reservoir called a tundish. This tundish is the starting point of the casting process, which is shown in Figure 7.

The molten metal flows from the tundish into the mold through the so-called submerged entry nozzle. The flow rate through this nozzle is controlled by either a stopper rod or a sliding gate. The mold is water-cooled. As a result, the liquid metal begins to cool down and take the shape of the mold. This creates a strand that is later pulled out through water-cooled rollers until the solidification process is fully complete [77]. Despite the relative simplicity of this process, it is not so straightforward that a good quality product is obtained.

Flow phenomena in the mold are complex, and they have a significant effect on the final product quality [78]. However, the classical non-tomographic measuring techniques cannot provide information about these flow patterns. For this reason, the problem of controlling the continuous casting process is usually reduced to controlling the mold level. This level can be measured, and it is known that its fluctuations have detrimental effects on product quality. Generally, this level should be as flat as possible to provide a smooth shell formation of the solidifying metal. Therefore, the literature on continuous casting control usually focuses on mold level only [79,80]. A variety of methods, including fuzzy control and model-based control, has been introduced, but the principal limit remains. No substantial control advancements are possible without tomographic sensing because the necessary information remains unavailable to the control system.

If desirable flow characteristics are to be achieved, it is necessary to extend not only the measuring equipment but also the actuating equipment of the process. Enhanced control over the flow in the mold can be achieved by using electromagnetic actuators besides the standard stopper rod. These actuators can be either electromagnetic stirrers or electromagnetic brakes. Stirrers create a rotating magnetic induction field, while electromagnetic brakes generate a static magnetic field to modify the fluid motion in the mold. Real-time measurement of these phenomena is not possible using conventional sensors. For this reason, electromagnetic actuators are normally operated as open-loop devices. One of the rare exceptions where electromagnetic actuators are used in a closed-loop arrangement is the research described in [81]. However, it should be noted that the configuration proposed in this paper uses sensing, which is very similar to tomography. The flow patterns in the mold are observed using a multitude of temperature sensors distributed over the mold plate (more than 2500 sensors). The flows are estimated based on the sharp thermal image of the mold obtained from these sensors.

Observation of the flow in the mold of a continuous casting machine was already mentioned in Section 2 of this paper when discussing tomography data processing and alternatives to computationally demanding and ill-conditioned image reconstruction. It is very important to achieve the desirable double roll flow pattern as shown in Figure 1a, while several numerical and controllable characteristics can be used to assess whether it was really achieved. The first such characteristic is the jet flow angle as shown in Figure 1b. Further characteristics, as described by Abouelazayem et al., in [41,44,82,83], include meniscus velocity, jet velocity, and jet impingement point.

All of these characteristics can be obtained from raw measurement data using parametric modeling, i.e., avoiding the image reconstruction step. The manipulated variables can be stopper rod position, electromagnetic brake current, or both. The cited papers used several configuration variants considering both SISO and MIMO control loops. The common feature was the use of models identified on the basis of measured data. These data were obtained from a small-scale continuous casting facility called Mini-LIMMCAST located at Helmholtz-Zentrum Dresden—Rossendorf. The description of this facility can be found in [44].

In all cases, a satisfactory modeling quality could be achieved with models of low order up to three. Mostly linear models were used; the most substantial non-linearities were modeled using Wiener models where static nonlinearity was expressed either by simple analytical function or using a neural network. Unlike previously mentioned applications of MPC, these papers used fully-fledged MPC, including all necessary constraints considering both limited magnitudes and rates of change of both the stopper rod position and electromagnetic brake current. The control based on UDV measurements has recently been extended to using contactless inductive flow tomography [84].

#### 3.3.4. Control of Hydrocyclone Separators

Control of hydrocyclone separators, which use somewhat similar concepts as the separator process described in some detail in Section 3.1.3, was also considered using PID controllers and electrical impedance tomography in the position of the measuring device. Such application is described in [85], while some preliminary concepts were outlined already in [86,87]. The controlled process is the solid–liquid separator. The proposed control system uses the fact that air-core size can be correlated with separation efficiency. Hence air-core size obtained from the electrical impedance tomography image is used as a controlled variable. The authors propose the use of a digital PID controller with a filtered derivative for this process while the tuning is left to experimental methods. Although paper [85] published in 2008 is based on a relatively long line of publications spanning more than one decade, the research presented there does not seem to have found any real continuation.

### 3.4. Knowledge-Based Control, Fuzzy Logic and Artificial Intelligence Approaches

As can be seen from the previous sections, in many cases, control with tomographic sensors is essentially real-time quality control, and its objectives are rather qualitative than quantitative. For instance, we need to achieve a good quality product from continuous casting or good separation efficiency. On the other hand, standard control assumes a specified set-point and feedback control loop that aims to have controlled variable(s) equal to set-point(s). Qualitative requirements must be first translated into standard control engineering terms and control objectives. In this regard, there is no difference between conventional PID control and advanced model-based control approaches such as MPC.

On the contrary, approaches based on fuzzy logic, artificial intelligence, and similar methods can be less rigid in their structure and specifications of objectives. Moreover, they can be designed and implemented without accurate mathematical models. This point is crucial. It is evident that even very simplified mathematical models of most of the processes considered here are very complicated.

Fuzzy control may be briefly introduced as an example of a control approach that can be used even if the process model is unknown or so complicated that controller design based on it would be totally impractical. Just the main ideas can be outlined in this paper. However, there are many good textbooks that can be used for further reference, e.g., recent titles [88,89].

It is well known that even very challenging processes can be controlled by human operators using their experience and expert knowledge. For instance, automatic systems cannot safely control aircraft takeoff and landing, but human operators (pilots) can handle even these flight phases. A fuzzy control system can be understood as a system implementing such expertise of a human operator. This expertise is not represented by differential equations or controller parameters but rather by situation/action rules. The operators are experts in operating their processes, and they know what to do under various circumstances. However, their knowledge is not expressed in precise rules such as, e.g., if the temperature is above 80 °C, set the heater power to 1 kW. Their rules are rather qualitative and imprecise. If we consider a really simple control task such as temperature control, we can say that the operator knows what to do, e.g., if the temperature is decreasing fast or if it becomes too high, while the term too high does not simply mean just higher than a single specific threshold.

Fuzzy rules are expressed in the same way as the rules in the minds of human operators. That means they are articulated using qualitative linguistic variables. Examples of typical fuzzy rules can be defined in the following way:

If the temperature is high and increasing, then reduce heater power fast.

If the temperature is high and constant, then reduce heater power slowly.

**Figure 8:**
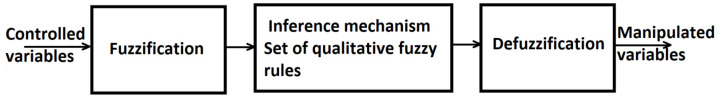
General structure of a fuzzy controller.

When fuzzy control is used for standard control tasks, it must be further considered that a fuzzy controller is similar to a human expert operator also in the sense that it looks at exact measured data, and if it performs a control action, this action is precisely specified. The measured temperature is a single numerical value, and the power of an electric heater can always be set only at one specific (crisp) value. It cannot be just low or high. For this reason, the fuzzy inference mechanism consisting of qualitative fuzzy rules must be connected to the real world by operations called fuzzification and defuzzification. This structure is shown in Figure 8.

**Figure 9:**
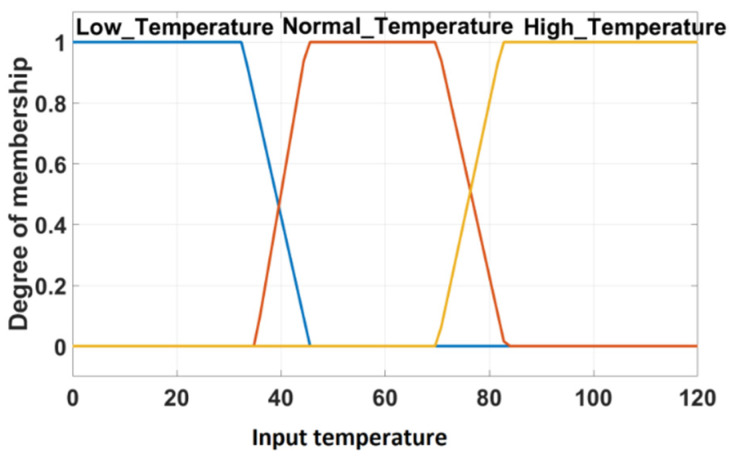
Example of an input membership function for temperature control.

The input to the fuzzification block is the measured values of controlled variables, e.g., temperature. Fuzzification means conversion to qualitative variables. It is done using the so-called membership functions, which convert the measurement into a degree of membership. An example of such a membership function is shown in Figure 9.

Assuming that the measured temperature is, e.g., 80 °C, this temperature is normal to a degree of 0.2, high to a degree of 0.8, and low to a degree of 0, where 1 represents full membership and 0 no membership. This expresses that in the considered control task, the human expert would regard 80 °C to be well above normal operating temperature. That means this temperature is definitely not low, rather high than normal, but on the other hand, the temperature could become even higher. Membership functions conceived in this way correspond to qualitative human reasoning where no strict boundaries exist between qualitative notions such as normal, high, too high, etc. The membership function is based on simple “expert” knowledge in this example. Defining these functions and inference rules in more complex tasks may not always be easy. For this reason, fuzzy control is also sometimes connected to other artificial intelligence approaches, especially with neural networks into neuro-fuzzy control [90].

**Figure 10:**
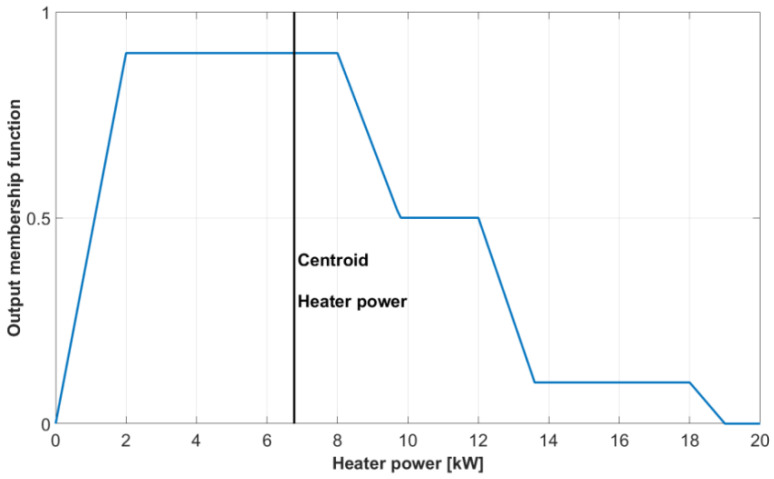
Defuzzification example resulting in a single numerical value of manipulated variable (heater power).

The output from the fuzzy inference mechanism combines all fuzzy rules together, and it is again a qualitative variable described by its membership function. Obviously, this function is not constant, but as the controlled variable(s) vary, fuzzy inference yields different results, and this output membership function also varies. This output must be defuzzified, i.e., converted to an exact numerical value that can be sent to the physical actuator, (e.g., heater). Defuzzification can be done in several ways. The most widely used method is the so-called centroid defuzzification which calculates the center of gravity (COG) of the membership function corresponding to the output variable along the x-axis. This defuzzification approach can be expressed by the formula

(25) $$\mathrm{COG}=\frac{\int_{a}^{b}\mu_{A}\left(x\right)xdx}{\int_{a}^{b}\mu_{A}\left(x\right)dx}$$

where *μ_A_* is the output membership function defined in the range from *a* to *b*. An example of this defuzzification method for one specific output membership function is shown in Figure 10.

The example with temperature control is a simple standard control task where precise temperature measurement must be fuzzified. An output from a tomographic sensor often gives directly qualitative information, e.g., a flow pattern. Although various flow patterns are clearly different if fully developed, the transitions between them may often be gradual and hence naturally fuzzy. For this reason, fuzzy control may be even more suitable for complex tomography-based control tasks than for simple standard control. Therefore, it is not surprising that control approaches from this category have been considered since the very beginnings of process tomography. An important application area has been pneumatic conveying, i.e., a process where an air (or other inert gas) stream is used as a transportation medium to transport various granular solids and dry powders. The most common variant of this process is the dilute phase pneumatic conveying where the conveyed particles are uniformly suspended in the gas stream.

This process has many advantages in terms of routing flexibility and low maintenance costs. However, an appropriate flow regime with a homogeneously dispersed flow must be continuously maintained. Otherwise, several issues may arise such as discrete plugs of material, rolling dunes with a possible high increase in pressure and a blockage, and unstable flow with violent pressure surges resulting in increased plant wear and product degradation. For this reason, standard uncontrolled pneumatic conveying processes can normally be used only for the specific solid materials for which they were designed, and airspeed must be rather high to avoid potential blockage problems. This high speed increases power demands and makes this process less energy efficient than it could be [91].

This is a motivation for closed-loop control. It is a non-standard control task. The control objective is to maintain a suitable flow pattern. There is no numerical-valued controlled variable with a specified set-point. Following [92,93], it is possible to identify several flow regimes besides the desired dilute phase flow achieved at a higher airspeed. If the airspeed becomes smaller, it reaches a point (so-called saltation velocity) where an abrupt change from dilute to dense phase flow occurs and the solid particles begin to settle out. This is marked by saltating flow and dune formation. If the speed is further decreased, so-called dune flow and plug flow can exist, and a significant danger of pipe blockage occurs.

The control of pneumatic conveying was treated by several research groups. The measurement system was always an ECT sensor, but different ECT data processing and control approaches were used. An earlier paper [92] by Deloughry, Ponnapalli, and others attempts to interpret the task of keeping the desirable flow regime as a standard control task with numerical-valued variables. It considers an experimental pneumatic conveying process where polyethylene nibs are transported. The tomographic image of the pipe cross-section has a total of 816 pixels, and the number of pixels that contain these polyethylene nibs (pixel density) can be used as a measure of the current level of sedimentation (dune formation) inside the pipe. This allows the use of a conventional PID controller whose set-point is set to a sufficiently small number to prevent dune formation. This objective was achieved with set-point 20, while set-point values higher than 50 usually resulted in pipe blockage. A substantial part of this paper discusses the influence of the PID controller parameters on the controller’s ability to prevent dune formation and blockage for different set-point values.

In the end, PID control was not found to be an adequate approach, and later the authors of [92] proposed a neural network inverse model controller [93]. ECT image was evaluated in the same way, i.e., by counting the pixels. The authors of [93] used the procedure of inverse plant identification [94]. That means they performed a wide range of experiments with the laboratory-scale pneumatic conveying process around the critical saltation speed. Data obtained from these experiments were first used to train a neural network modeling the process in forward configuration, i.e., current and past values of air blower speed and past values of pixel density were inputs, while the current value of pixel density was output.

**Figure 11:**
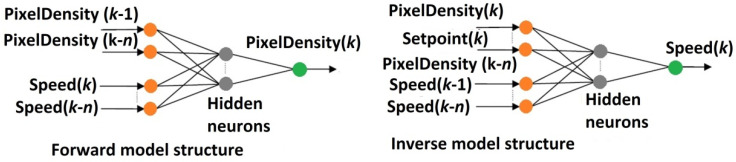
Schematic of the forward and inverse neural model (inverse model = controller).

The purpose of the forward model was to find suitable neural network parameters (number of hidden neurons and past values). The network with the parameters that turned out to be the best for modeling the process (6 hidden neurons and 13 past values) was then trained in the inverse configuration where the current value of the blower speed (i.e., manipulated variable) is the output. Since the output of this model is the manipulated variable, and the set-point can be fed into the current pixel-density input, this neural inverse model can be understood and used as a controller. These model structures are illustrated in Figure 11.

The paper concludes that this controller was able to clear the dunes automatically while maintaining the air velocity at a minimum value necessary to keep the flow homogeneous. In this respect, it was able to outperform the PI/PID controller. This neural controller responds in some way to changes in flow patterns, but this response is implicit and hidden in the internal structure of the network. There is no direct and explicit identification of these regimes.

An alternative approach is followed by Williams, Owens, and others in [91]. It uses an explicit classification of flow regimes. The ECT data are processed to obtain the void fraction, i.e., a signal that can be considered complementary to the pixel density, which evaluated the number of occupied pixels. Both magnitude and frequency of changes of void fraction signal are important. Classification of flow regimes is performed by a neural network. The authors then develop an idea of a two-level control strategy. Low-level control with a very fast response does not use ECT measurements. Only flow rate and pressure measurements are used to quickly prevent any blocking tendencies.

High-level control is based on fuzzy logic, and it uses flow classification from neural networks and data from other sensors to keep the desired flow regime and retune the low-level controller. The classification of flow regimes seems to be really tested and working. On the contrary, the control structure proposed by the authors is rather a concept, which is proposed, but not really implemented. This concept does not seem to be ever used for control of the pneumatic conveying process. Papers [93,94] are later (2001 and 2007), and they use a different approach without even citing [91] (1999). Despite this, the approach taken in [91] turned out to be pretty fruitful and even paradigmatic for controlling other processes. Its main principle can be characterized as the classification of the selected phenomena (in this case, flow patterns) in the tomography data as a first stage and fuzzy or other rule-based systems as a second stage in the control strategy.

This concept is used in [95] to control oil separators based on ECT images. The ECT images are classified using the principal component analysis approach. The results from this classification are then used as input to a controller based on expert rules. This controller was applied to a laboratory-scale separator process. In Section 2 of the present paper, we have already mentioned paper [40] where the classification of flow patterns was done using both neural networks and cascades of Support Vector Machines. These classified patterns were then used as inputs to model-free adaptive controllers based on deep belief networks. Model-free adaptive control [96] is a control approach that might be very suitable for use with tomography because, as we could see, the models are notoriously difficult to obtain. It is hard to tell how this approach is applied in [40] because the paper focuses mostly on the classification of flow patterns while the control itself is only outlined. Nevertheless, it is easy to see that the paper again follows the same structure: flow patterns classification using artificial intelligence methods as a first stage and fairly non-classical controller as a second stage.

A similar structure is followed also by [97], where the focus is on control of the polymer extrusion molding process. ECT tomography is used as the only sensor that can measure the internal temperature in a cross-section of a polymer extrusion process. In this way, ECT enables feedback control of this process normally operated by trial and error. The melt temperature field is obtained from the reconstructed ECT image measured. The difference between the measured and reference temperature fields is then used as input information for a knowledge-based control system. This system uses process variables such as temperature in different sections along the barrel and the screw rotating speed as manipulated variables. That means there is an implicit cascade control structure where this knowledge-based controller is the master controller calculating set-points for slave controllers in the polymer extruder. Similar to most of the previous papers cited in this section, this knowledge-based controller is a proposed concept but was neither implemented nor tested.

## 4. Discussion and Future Research Directions

A very recent survey paper on fast tomographic imaging techniques written by the leading experts in the field [4] concludes that the tomography hardware, as well as the standard image reconstruction and data processing methods, have by far reached at least the technology readiness level (TRL) 7 (System prototype demonstration in operational environment) in terms of process diagnostics. It is more than evident from the previous section that the development of closed-loop control applications of tomography lags significantly behind the development of hardware and data processing technology.

Considering the papers cited in the previous pages, there is no doubt that all of them correspond at least to TRL 2 (Technology concept formulated). However, some of them, and this is especially true of most of the papers mentioned in the subsection on knowledge-based control and artificial intelligence approaches, do not really go beyond formulating the concept, i.e., beyond TRL 2. Fortunately, other papers, and some of them in this special issue, tested the validity of their concepts using experiments either in simulation or with laboratory-scale processes. Hence, in general, we can say that the TRL of closed-loop control with tomographic sensors is somewhere between TRL 3 and 4 (Experimental proof of concept, Technology validated in a lab). Therefore, significant future research effort is still needed to make control with tomographic sensors a standard industrial technology.

Although true, such a conclusion would be too vague. Section 3 gives an almost complete list of papers on tomography applications for closed-loop control, allowing a more detailed analysis.

One of the most specific features of tomographic sensors is their ability to observe multidimensional space-distributed variables. This may lead to the conclusion that the most fruitful control concept is likely to be the connection with the automatic control methods for the control of distributed parameters systems. A significant part of published papers has chosen to go this way, and we discussed their results in Section 3.1. All of these papers decided to use the early lumping approach. PDEs describing the process were converted into high-order state-space models using FEM or similar methods. State-space models allow seamless connection with data reconstruction using state estimation and with many advanced control methods based on state-space models. As a whole, this approach exhibits a significant degree of mathematical elegance.

Despite this, it is uncertain whether this line of research can ever proceed to the stage of industrially applicable technology. In Section 3.1., we considered relatively simple processes, and the order of the state-space models was in the range of thousands. In the research done by the authors of the present paper, an attempt was made to apply the same approach to the continuous casting process with its complicated flow patterns [98]. Despite numerous simplifications, the order of the state-space model was in the range of tens of thousands. Implementation of control based on such models is virtually impossible because of ill-conditioning problems, and no future advances in computing power can change this principal issue.

In Section 3.1.4, we discussed the possibility of replacing FEM with spectral and other space-discretization methods using global spatial basis functions. These approaches result in relatively lower-order models. To the best of our knowledge, there is no paper that would use this class of approaches in connection with tomographic sensors. In spite of that, this may be one of the viable methods to respect the fact that tomographic sensors can sense distributed variables and that the underlying dynamics are a PDE system while keeping the model order low enough. Similarly, we can appreciate the approach described in Section 3.2. This approach is actually hybrid in a sense because a lumped dynamical model is connected with a static function modeling the distributed variable. Thus, only the control relevant distributed aspects of the process are kept, and the resulting model is simple enough to be used for control.

In 2018 an important and widely cited paper, “Applying industrial tomography to control and optimization flow systems”, was published [99]. Quite characteristically, it discusses almost exclusively tomography hardware and data processing methods. Nevertheless, it rightly demands that the concept of the process control based on tomography requires, among other developments, also a correct extension of the classical control theory because this theory is not sufficiently developed for a large amount of sensor data.

The approaches mentioned above, i.e., space-discretization methods using global spatial basis functions and hybrid approaches keeping only the most relevant distributed aspects of the process, may be one of the responses to this demand. It is important to note that it is a relatively transparent and mathematically consistent response, unlike black box and artificial intelligence approaches. On the contrary, FEM discretization of PDE models may be more straightforward and with larger support in software packages, but it is unlikely to produce useful control-oriented models. Similarly, we have mentioned in Section 2 that also late lumping approach exists, and it can be an alternative to early lumping methods. However, given its complexity, it may be an interesting niche for basic research, but at this moment, it is unlikely to be a suitable way to push tomography-based control to higher TRL levels.

Further, it is pretty clear that the research direction outlined above, though potentially fruitful, definitely is not a panacea. It assumes first-principles modeling as a first step. Even if we consider the famous quote: “All models are wrong, but some are useful”, we can say that many of the processes described above are so complex that finding mathematical first-principles models that would be at least somewhat useful is virtually impossible.

Thus, it is not surprising that most of the closed-loop control applications described above that were either experimentally tested or came close to experimental testing were based on models obtained through system identification. Even the hybrid model of fluidized bed dryer was partly identification-based because the values of coefficients in (17) were obtained experimentally. Usually, the papers used standard identification methods as available in the respective Matlab toolbox. However, the field of identification is constantly developing, and new, more powerful methods particularly suitable for the identification of state-space models appear (e.g., [100] and references therein). For these reasons, the use of identification-based models for control with tomographic sensors is not the last resort but potentially a fruitful research direction.

Models can be obtained in different ways. However, if we consider conventional (i.e., not fuzzy, neural, and similar models) mathematical models, it should be pretty clear from this paper that if an advanced model-based control is to be used, there is not much point in using LQ control and any other method than MPC. MPC is the only method that can respect all process constraints, and that is flexible enough to accommodate a wide range of various control objectives.

Perhaps the most surprising result from this review is the content of Section 3.4. It shows that artificial intelligence methods, fuzzy control, knowledge-based control, and other similar control methods are rarely connected with tomographic sensors. Moreover, most of the references that could be cited in this section are rather old. Mostly they describe research that started in the mid-1990s, was published in the first decade of the 21st century, and then did not continue further. This is unexpected considering how complex and not well amenable to modeling are many of the processes that can benefit from tomography-based control. Fuzzy modeling, neural networks, or model-free approaches such as [96] seem to be a natural answer to this situation. Model-free adaptive control [96] is very attractive because of its purely data-driven model-free feature. Its use in connection with process tomography was rather just mentioned than really implemented in [40] and the way to applications is likely to be longer. However, the use of neural networks and fuzzy approaches for control is now well established [101,102], and this research direction is now perhaps at the same time both the most underutilized one and the one most likely to bring relevant results in a not too long time.

The fact that such results are needed is beyond any doubt. Although the present paper focused primarily on automatic control methods, it has also shown many processes for which tomography-based control is beneficial. Beneficial is a general and ambiguous term. However, in this concluding part of the paper, it can be stated more precisely what should be understood under the term beneficial.

We reviewed several closed-loop control applications of tomographic sensors. Concentration distribution control was a simple generic example of how controller design based on the early lumping approach can be applied to processes governed by the convection-diffusion equation. However, all other applications were applications to important processes widely used in different branches of industry. Standard instrumentation of most of these processes includes a certain number of non-tomographic sensors and control loops. Comparison of control based on tomographic sensors with these conventional control loops is a complex question, and something such as direct comparison is virtually impossible. Tomographic sensors are not introduced to replace the conventional sensors and achieve more or less the same control objectives, perhaps somewhat better. Their purpose is to open new control perspectives and enable new objectives that were unthinkable with conventional sensors.

One example can be the continuous casting process discussed in Section 3.3.3. Conventional control is based on the requirement that the liquid level in the mold should be as stable as possible to guarantee that the formation of the solidifying shell will be smooth. That means conventional control can use more or less advanced and sophisticated methods, but it is still simply a mold level control based on a mold level sensor. As such, it has no access to the processes under the surface of the metal in the mold. On the contrary, tomography opens direct access to the flows in mold, and the control loop can be closed based on these measurements. The reviewed papers condensed the tomographic measurements into relatively simple numerical characteristics. This class of approaches is not the only option. It would also be possible to evaluate and classify the control patterns similarly as it was done with the pneumatic conveying process. In any event, regardless of which approach to working with tomographic measurements is used, tomography enables us to specify entirely new control objectives and control such aspects of the continuous casting process that are otherwise uncontrollable.

Similar conclusions can be drawn from the other processes described in this paper. Tomography-based control of the inline fluid separator can be much more direct than the control based on pressure drop ratio. The potential benefits of tomography-based control are even higher in the case of microwave drying. Process tomography is the only technology that can measure the moisture distribution inside the material (avoiding hot spots and ignition danger), while alternative sensor technologies provide only surface-related information. Hence, the main conclusion from this paper is not that conventional control objectives can be achieved better if tomography-based control is used, but that entirely new control perspectives can be opened by process tomography.

**Figure 12:**
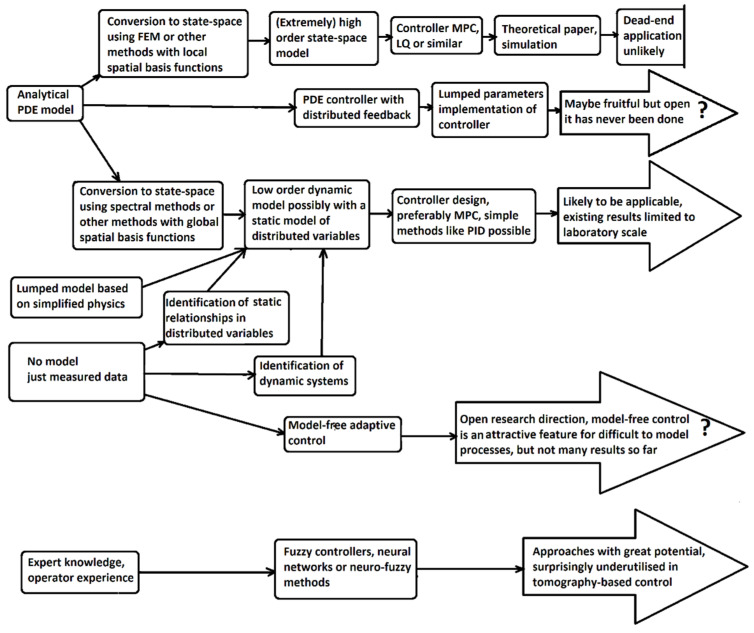
Potential of modelling and control approaches for tomography-based control.

The above discussion of the future potential of the various automatic control methods for tomography-based control is summarized in Figure 12. This figure starts on the left side with different models or, more generally, with different pieces of input information that can be used for controller design. Then it continues with various transformations of this input information that is necessary so that it can be used for controller design. On the right side, the future potential of each specific way from input information to closed-loop control is estimated.

## Data Availability

The study did not report any data.

## Abbreviations

ECT	Electrical Capacitance Tomography
EIT	Electrical Impedance Tomography
FEM	Finite Element Method
LQ	Linear Quadratic
ODE	Ordinary Differential Equation
PDE	Partial Differential Equation
PID	Proportional Integral Derivative
MIMO	Multi Input Multi Output
MPC	Model Predictive Control
SISO	Single Input Single Output
TRL	Technology Readiness Level
